# Using DEXA to diagnose impending atypical femoral fracture in an asymptomatic patient on long term bisphosphonates: A case report of a missed opportunity for fracture prevention

**DOI:** 10.1002/ccr3.9524

**Published:** 2024-10-23

**Authors:** Ashraf Amin Ariff, Panagiotis Konstantinou, Michael Cuss, Charmaine Riley Nelson, Ahmed Hamed, Anastasios P. Nikolaides

**Affiliations:** ^1^ NHS Foundation Trust Birmingham Heartlands Hospital, University Hospitals Birmingham Birmingham UK; ^2^ NHS Trust Royal Cornwall Hospital Truro UK

**Keywords:** atypical femoral fractures, bisphosphonates, DEXA, osteoporosis

## Abstract

We report a patient who sustained an AFF (Atypical femoral fractures) after 15 years of bisphosphonate treatment. DEXA scans can be utilized to identify early evidence of AFF in asymptomatic patients up to 9 years before the development of an AFF.

## 1 BACKGROUND/INTRODUCTION

Osteoporosis and associated fragility fractures are major contributors to morbidity and mortality globally.^1^ Oral bisphosphonates (BPs) are a mainstay for the management of osteoporosis and have had a significant role in reducing the disease burden.^2^ BPs act by inhibiting osteoclast activity to prevent bone resorption, reduce bone degradation and subsequent risk of fractures.^3^, ^4^ However, paradoxically, it is well‐documented that long‐term BP use increases risk of atypical femoral fractures (AFFs).^5^, ^6^

The American Society for Bone and Mineral Research (ASBMR) Task Force defines AFFs as atraumatic or minimally traumatic proximal femoral fractures arising from the lateral cortex.^7^ The ASBMR describe prodromal changes in the lateral cortices that can be recognized radiologically, known as “beaking” or “flaring”, caused by thickening of the lateral periosteum or endosteum. BP‐induced inhibition of bone remodeling by osteoblasts is thought to weaken the lateral cortices and contribute to the accumulation of micro‐damage, making the bone susceptible to AFFs.^4^ Patients with impending AFFs can have prodromal symptoms including hip, pelvic or proximal leg pain.^8^ In these patients, BP treatment is withheld and proximal femur X‐rays are used to identify any radiological evidence of complete or incomplete AFF. If identified, BPs are discontinued and alternative pharmacological therapy, such as teriparatide alone or combined with operative treatment may be indicated. Teriparatide is a recombinant human parathyroid hormone which increases the bone mineral density (BMD) of the lumbar spine and femoral neck in patients with postmenopausal osteoporosis.^9^ The primary operative management option for acute AFF is Intramedullary (IM) nail fixation with Cephalomedullary femoral nailing (CMN).^10^

This is a case presentation of a patient who sustained bilateral AFFs after long‐term BP treatment. It demonstrates that radiological evidence of these impending fractures was identifiable on routine DEXA scans, 9 years prior to the current admission.

## 2 CASE HISTORY/EXAMINATIONS

A woman in her 80s presented with left hip and knee pain after a fall. She fell backwards after accidentally bumping into her husband when passing him on the landing of their home, also sustaining a minor head injury. An ambulance was called as she was unable to weight bear. On admission to the emergency department, clerking doctors noted her left hip was shortened and externally rotated. She remained in pain, but was orientated and observations were stable.

Prior to admission she was independently mobile, and walked comfortably upstairs to her bedroom, although she occasionally walked with a stick outdoors. She lived with her husband, needing no help from carers, was a non‐smoker and occasionally drank small amounts of alcohol when socializing.

She had a past medical history of osteoporosis and had been on 70 mg alendronic acid weekly for a total of 15 years. The drug was stopped 4 months prior to admission due to the long duration of treatment. She had routine DEXA scans at 9 years and 7 years before admission, which showed normal femoral BMD on both occasions. She sustained a pelvic fragility fracture 4 years previously, which was managed conservatively. A routine DEXA scan completed 7 months before the current admission showed femoral osteopenia (T‐score − 1.2). Her other co‐morbidities included a previous breast cancer, type 1 diabetes mellitus, Coeliac disease, hypertension, chronic kidney disease, gout, atrial fibrillation, for which she was on anticoagulation, and she had a percutaneous pacemaker fitted.

## 3 INVESTIGATIONS

**FIGURE 1:**
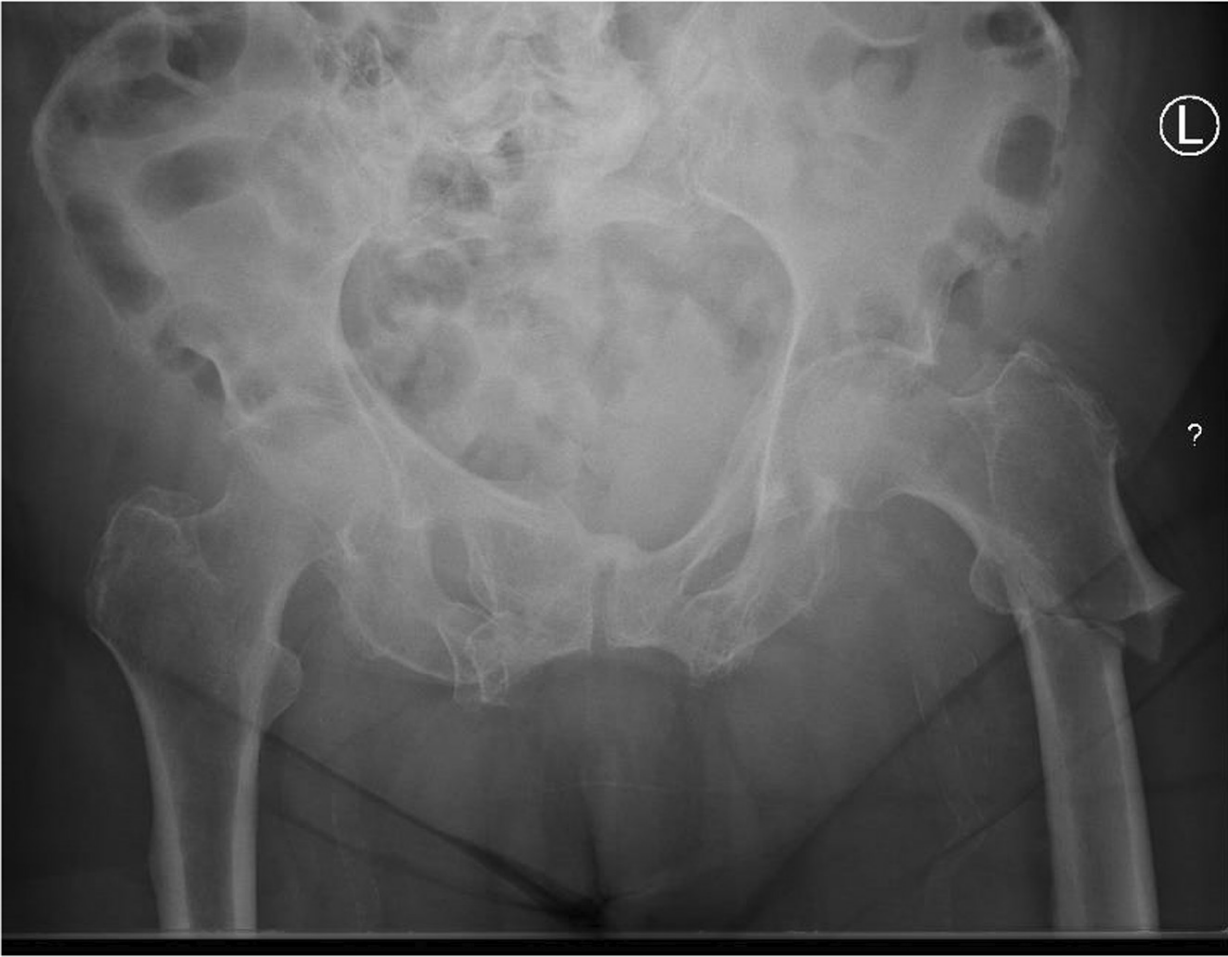
Pelvic X‐ray on day of admission: Acute transverse subtrochanteric fracture of the left neck of femur with medial displacement. Contralateral cortical thickening at the lateral aspect of the right subtrochanteric region was noted.

Admission bloods demonstrated a raised creatinine kinase and mildly raised inflammatory markers. Her vitamin D and endocrine laboratory findings, including calcium, alkaline phosphatase and thyroid hormone levels were within the normal range. An initial pelvic X‐ray identified a left atypical subtrochanteric fracture, and contralateral subtrochanteric lateral cortex thickening (Figure 1).

## 4 TREATMENT

The patient's complete subtrochanteric AFF was treated operatively the following morning with IM nail fixation using a cephalomedullary nail (CMN) on a traction table. Prophylactic CMN of the contralateral right femur was planned for a later stage, once the patient had recovered from the acute fracture, given the patient's frailty and past medical history, as the patient did not mention clinical signs of impending atypical femoral fracture on the right side at that time. Postoperatively, the patient was permitted to bear weight as tolerated and underwent regular physiotherapy. According to our physiotherapy protocol, we encourage weight bearing based on postoperative reduction, unless strong radiographic evidence indicates a gap between fragments or varus reduction. The patient should visit our outpatient fracture clinic every 6 weeks for standard postoperative examinations, including imaging studies. Bone union is considered achieved when new bone formation over the fracture line is confirmed on frontal and lateral plain X‐ray films. Orthogeriatric review was sought, with the view to start teriparatide for ongoing osteoporosis management.

## 5 OUTCOME AND FOLLOW‐UP

**FIGURE 2:**
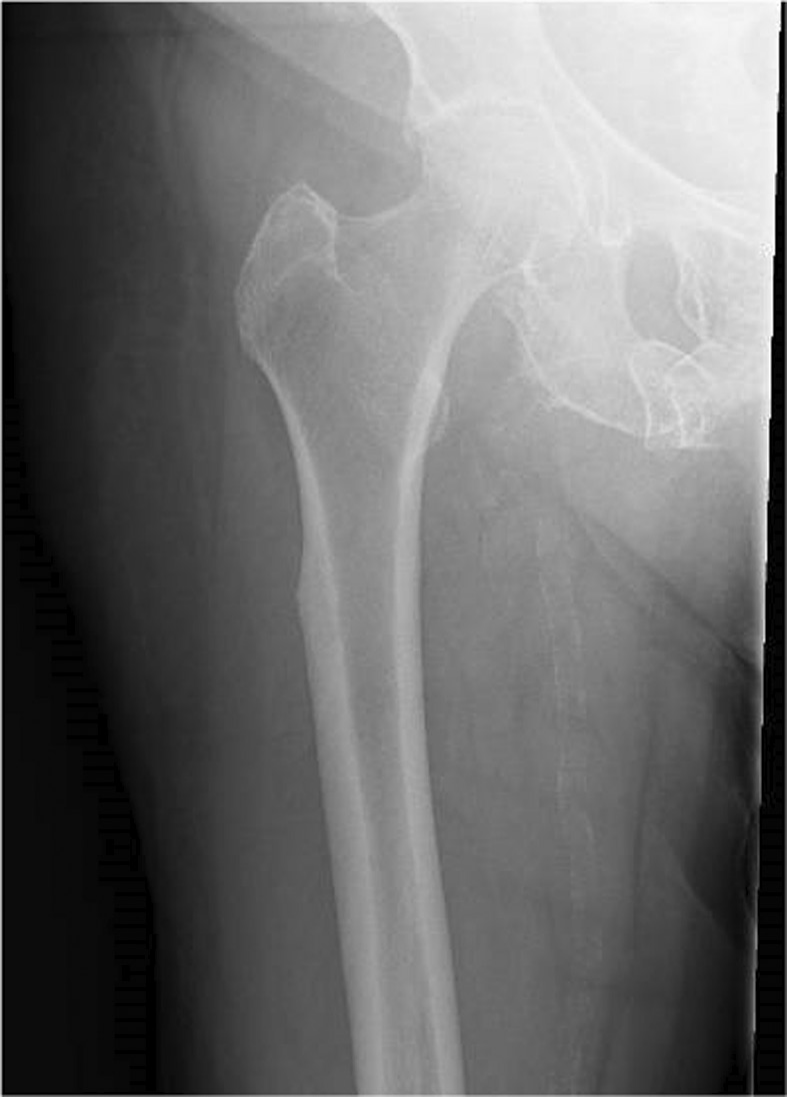
X‐ray right femur—Beaking of the subtrochanteric lateral cortex with faint sclerotic changes in the adjacent trabeculae indicate an impending AFF.

**FIGURE 3:**
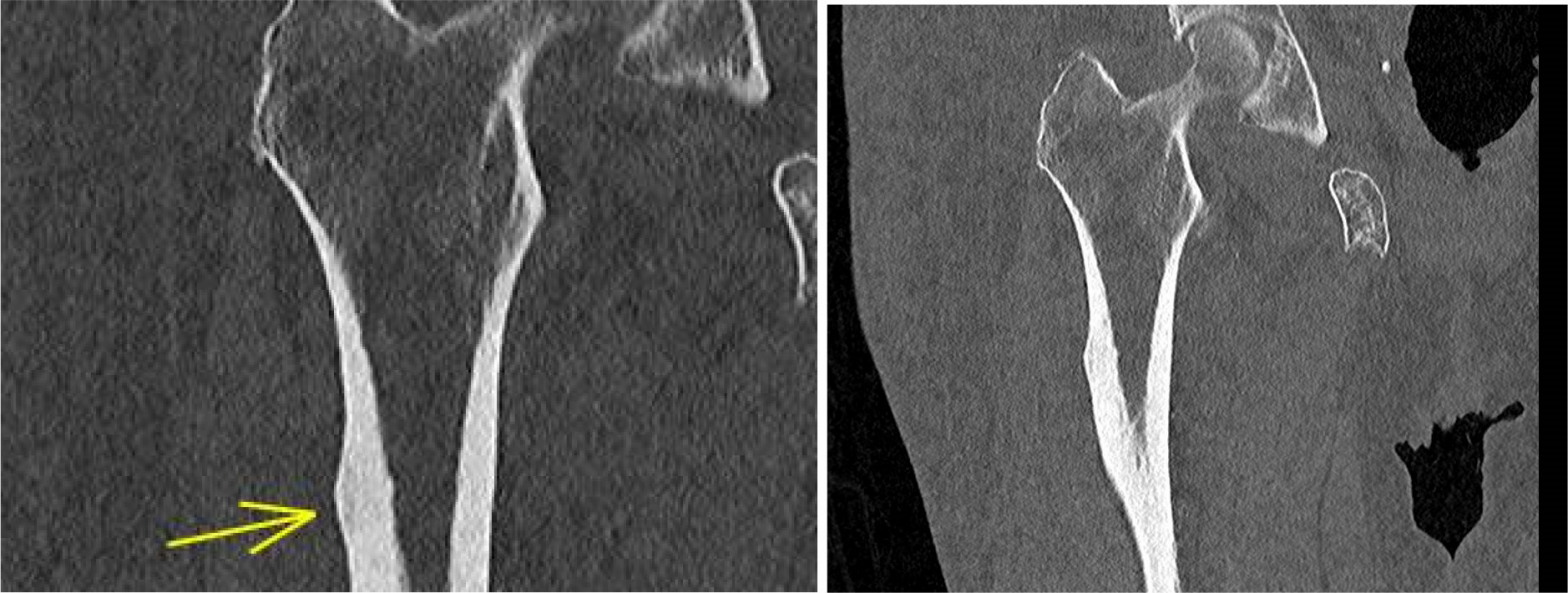
CT right lower limb—Focal cortical thickening along the lateral aspect of upper metaphysis of right femur, with subtle linear hypodensity within. Cortical thickening was also noted in the medial aspect of the subtrochanteric region. However, no evidence of any obvious cortical breach or fracture noted.

The patient was transferred to a local rehabilitation unit and progressed well with physiotherapy, although there were persistent issues with glycaemic control. She then developed new right sided thigh pain, and was re‐admitted to an acute ward with diabetic ketoacidosis (DKA). A right femur X‐ray highlighted previously identified lateral subtrochanteric cortical thickening, with a subtle sclerotic line traversing the cortex, indicating a possible AFF (Figure 2). A Computerized Tomography (CT) scan of the right lower limb ruled out an acute fracture, but further demonstrated beaking of the left lateral cortex, and noted cortical thickening in the medial aspect of the subtrochanteric region (Figure 3).

**FIGURE 4:**
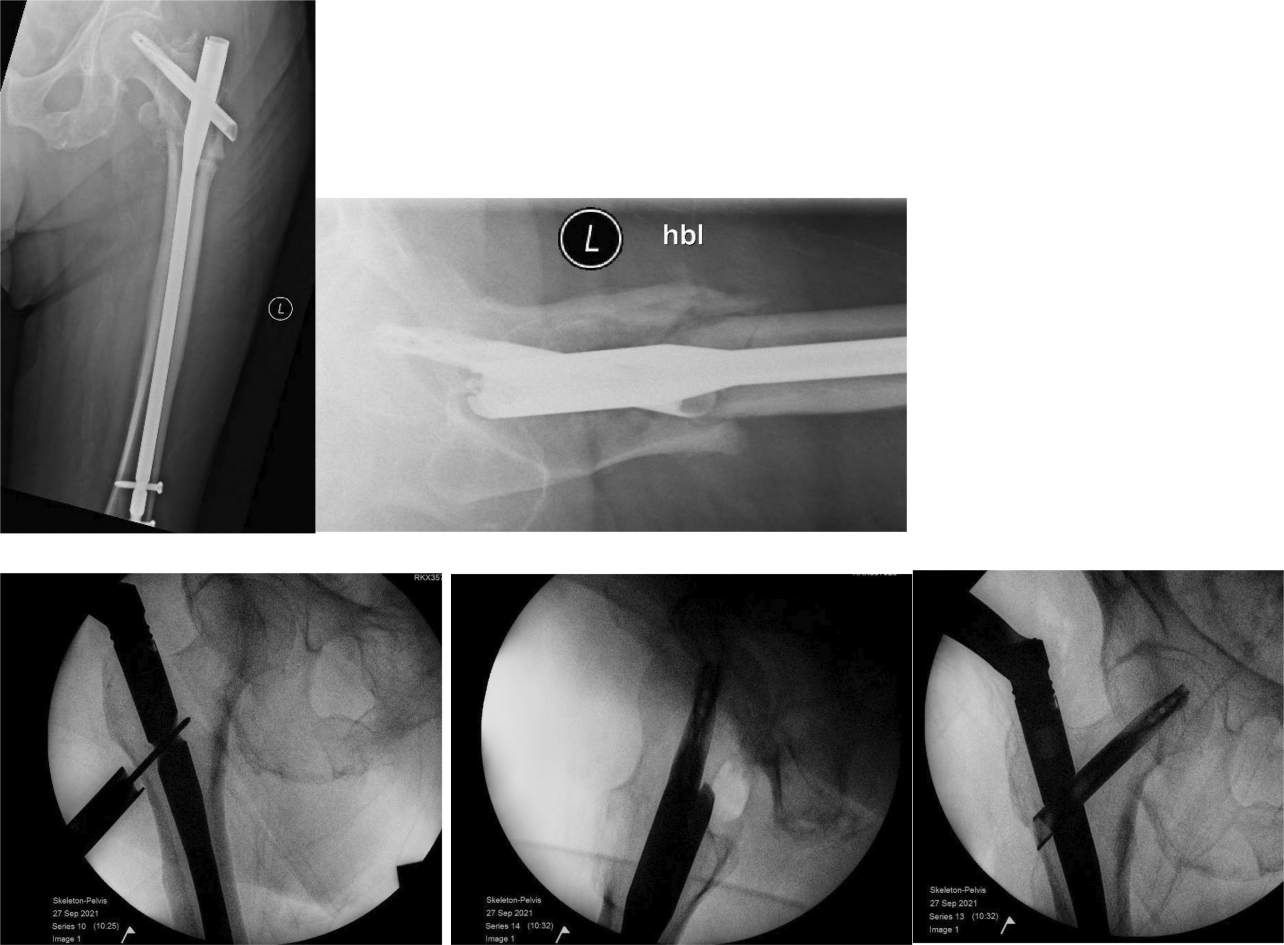
Postoperative X‐rays of left femur and intraoperative fluoroscopy images of right femur.

Treatment options were discussed with the patient. Given the acute onset of right hip pain and high risk of complete AFF, prophylactic right PFN was agreed upon. Surgery took place 25 days after the fixation of the contralateral complete AFF. Postoperative and intraoperative radiographic images of both surgeries are shown in Figure 4.

Post operatively the patient developed an acute kidney injury (AKI), but was otherwise lucid and well. She was then transferred to a local rehabilitation hospital on the second post operative day. Her AKI improved with intravenous (IV) fluids, and she received IV ferinject for a microcytic anemia (Hemoglobin 76). Preparations for discharge were made following good progress with physiotherapists, and follow up with the diabetic team was organized. One week post‐operatively, the patient was found unresponsive in bed and resuscitation attempts were initiated. Her blood gas demonstrated profound hypoxia, raised lactate and respiratory acidosis. Despite resuscitation attempts, the patient had a persistently non‐shockable rhythm. After 25 minutes of cardiopulmonary resuscitation (CPR), the decision was made to stop treatment and the patient passed away. Her death was attributed to hypertensive cardiac disease, agreed by coroners without further investigation.

## 6 DISCUSSION

There is a well‐established link between long term BP therapy and risk of AFFs.^4^, ^7^, ^11^, ^12^ As described in the 2014 ASBMR report,^7^ evidence suggests that AFFs arise from stress fractures. Stress fractures result from the accumulation of microscopic cracks in cortical bone, which develop over time and result in the development of a lateral cortical “bump.”^13^ Bones subjected to repetitive loading that exceeds the body's capacity for repair are at risk of these fractures. In normal bone physiology, areas with microscopic cracks are resorbed by osteoclasts and replaced with new bone by osteoblasts, a process known as targeted modeling. However, BP‐induced inhibition of bone remodeling by osteoblasts is thought to weaken the lateral cortices, thereby increasing the risk of fracture development from these areas.^4^

The ASBMR categorize the AFF in terms of major and minor features, outlined in Table 1. They state that AFFs must originate “along the femoral diaphysis from just distal to the lesser trochanter to just proximal to the supracondylar flare.” Our patient had 4 major features (1, 2, 4, and 6) and 2 minor features (2 and 3) on her right femur.

**TABLE 1:** American Society for Bone & Mineral Research (ASBMR) major and minor features of atypical femoral fracture.^7^

Major Features: (4 out of 5 features must be present)	Minor Features: (None required, but have sometimes been associated)
Fracture is associated with minimal or no trauma (i.e. Fall from a standing height or less) Fracture line originates at the lateral cortex and is substantially transverse in its orientation (although it may be oblique as it progresses medially across the femur) Complete fractures: extend through both cortices and may be associated with a medial spike Incomplete fractures – involve only the lateral cortex Non comminuted or minimally comminuted fractures Localized periosteal or endosteal thickening of the lateral cortex is present at fracture site (beaking or flaring)	Generalized increase in cortical thickness of the femoral diaphysis Unilateral or bilateral prodromal symptoms (dull/aching pain in groin/thigh) Bilateral incomplete or complete femoral diaphysis fractures Delayed fracture healing

In the last decade prescription of BPs has fallen, attributed in part to negative media coverage on risks of the treatment, and Federal Drug Agency (FDA) announcements on possible side effects.^6^, ^14^ Patients on oral BPs for more than 5 years should routinely be reassessed to determine the need for ongoing treatment.^15^ DEXA scans quantify BMD and are used routinely to diagnose and monitor osteoporosis.^16^ Osteoporotic fracture risk is assessed using the Fracture Risk Assessment Tool (FRAX), which calculates the 10‐year probability of osteoporotic fractures. If fracture risk is low, patients can undergo a “drug holiday”, where BP therapy is paused temporarily or indefinitely. Drug holidays are indicated after 4–5 years of treatment in patients with medium risk of fracture, and 10 years in those at high risk.^17^ In our case, the patient was on alendronic acid for a total of 15 years and was therefore at a higher risk of developing complications including AFF. However, research has shown that patients on a drug holiday can have an increased risk of hip fracture.^18^ It is therefore important that the risks of discontinuing BP therapy are weighed up against the risk of AFF.

### 6.1 Management of AFF

The ASBMR treatment summary for AFF (Table 2) advises a trial of conservative management for asymptomatic impending AFF, and prophylactic operative treatment in those presenting with pain.^7^

**TABLE 2:** American Society for Bone & Mineral Research management recommendations for atypical femoral fracture.^7^

Management of AFF
For patients with a stress reaction, stress fracture, or incomplete or complete subtrochanteric or femoral shaft fracture, potent antiresorptive agents should be discontinued. Dietary calcium and vitamin D status should be assessed, and adequate supplementation prescribed. Prophylactic reconstruction nail fixation is recommended for incomplete fractures (with cortical lucency) accompanied by pain. If the patient has minimal pain, a trial of conservative therapy, in which weight‐bearing is limited through the use of crutches or a walker, may be considered. However, if there is no symptomatic and radiographic improvement after 2 to 3 months of conservative therapy, prophylactic nail fixation should be strongly considered, because these patients may progress to a complete fracture. For patients with incomplete fractures and no pain, or those with periosteal thickening but no cortical lucency, limited weight‐bearing may be continued and vigorous activity avoided. Reduced activity should be continued until there is no bone oedema detected on MRI or no increased activity detected on bone scan.

Incomplete fractures can progress into a complete fracture with low energy trauma or without extrinsic trauma. This can make surgery more challenging, result in higher complication rates and poorer clinical outcomes.^19^, ^20^ Furthermore, evidence shows that a non‐operative treatment of BP‐related femoral stress fractures is unreliable, as the majority progress to fracture completion, and performing a prophylactic PFN also reduces total hospital admission time.^21^

**FIGURE 5:**
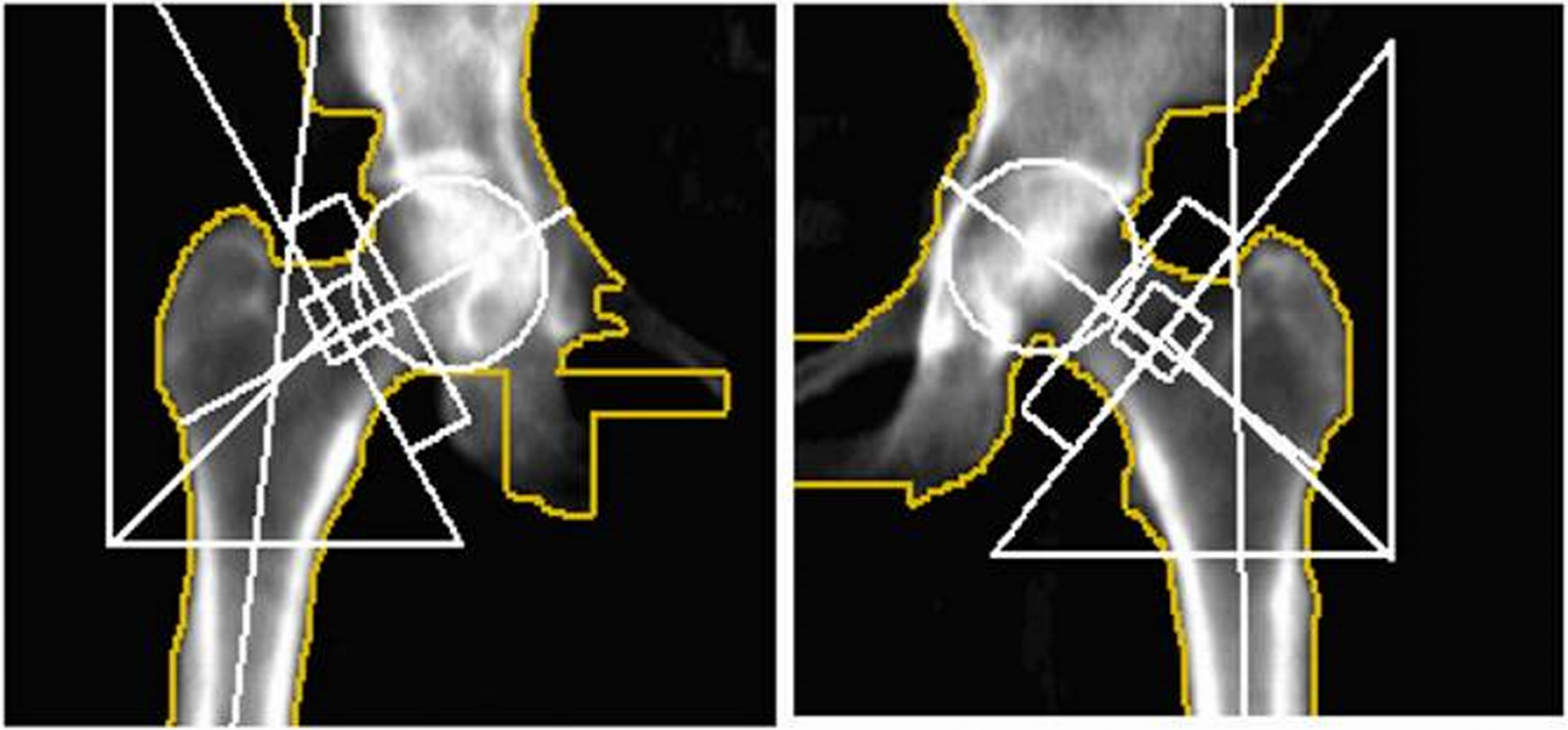
DEXA scan 9 years prior to admission showing bilateral beaking of lateral cortex.

**FIGURE 6:**
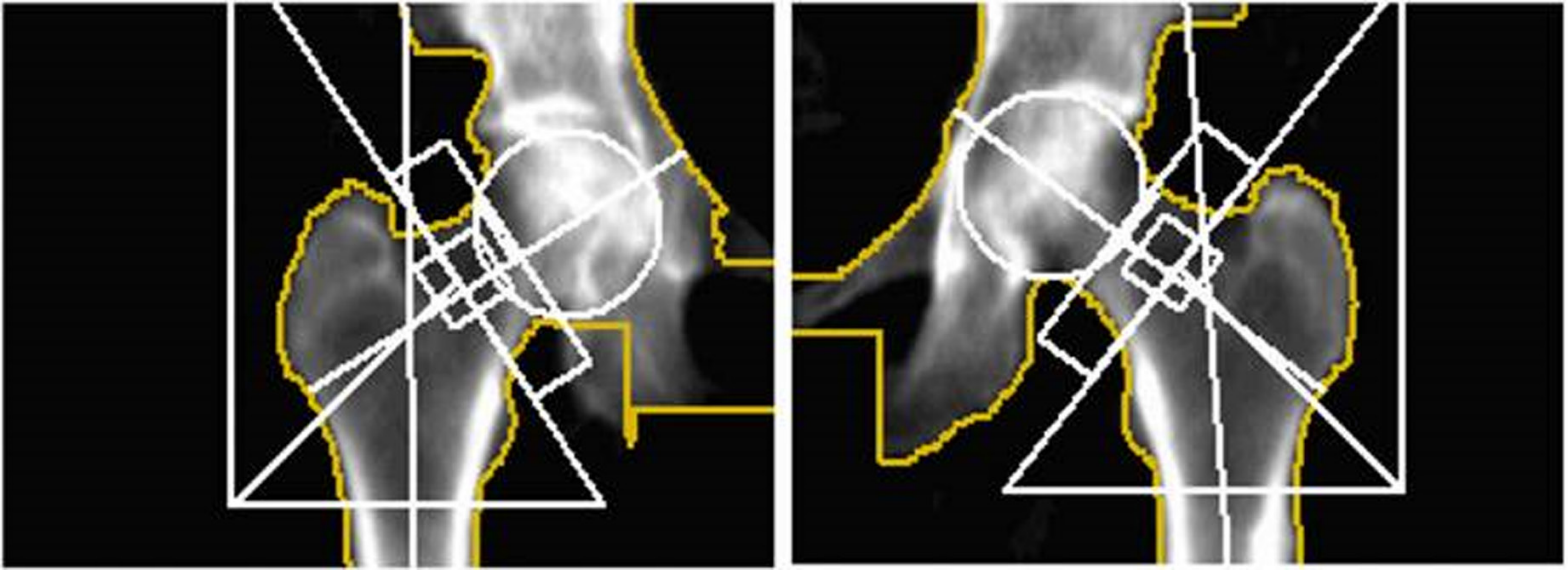
DEXA scan 7 years prior to admission showing progression of beaking in the left femur lateral cortex.

Previous studies have highlighted the value DEXA scans in identifying impending AFFs in patients on BP therapy. They conclude that DEXA scans should be used as a screening tool, in order to prevent progression to complete AFF.^22^, ^23^, ^24^, ^25^ Both DEXA and plain radiography are based on X‐rays; however, DEXA is already included in the follow‐up of patients treated for osteoporosis. Physicians can take advantage of this examination to detect impending AFF before clinical signs appear. Additionally, the radiation exposure from DEXA is very low, the method is cost effective, and it is preferable to use this examination as it provides data on bone density as well as detecting impending AFF.^26^, ^27^ A retrospective review of the patients routine DEXA scans at 9 years prior to acute admission demonstrated bilateral cortical thickening of the lateral cortices (Figure 5). T scores were normal (left femur T‐score 0.1, right femur −0.6, mean femur T‐score − 0.3). Repeat DEXA scans carried out 2 years later show subtle progression of beaking in the lateral cortex of the left femur (Figure 6). Femoral bone density had fallen since the initial DEXA, however femur T‐scores remained normal (left femur T‐score − 0.2, right femur −0.9, mean femur T‐score − 0.6).

**FIGURE 7:**
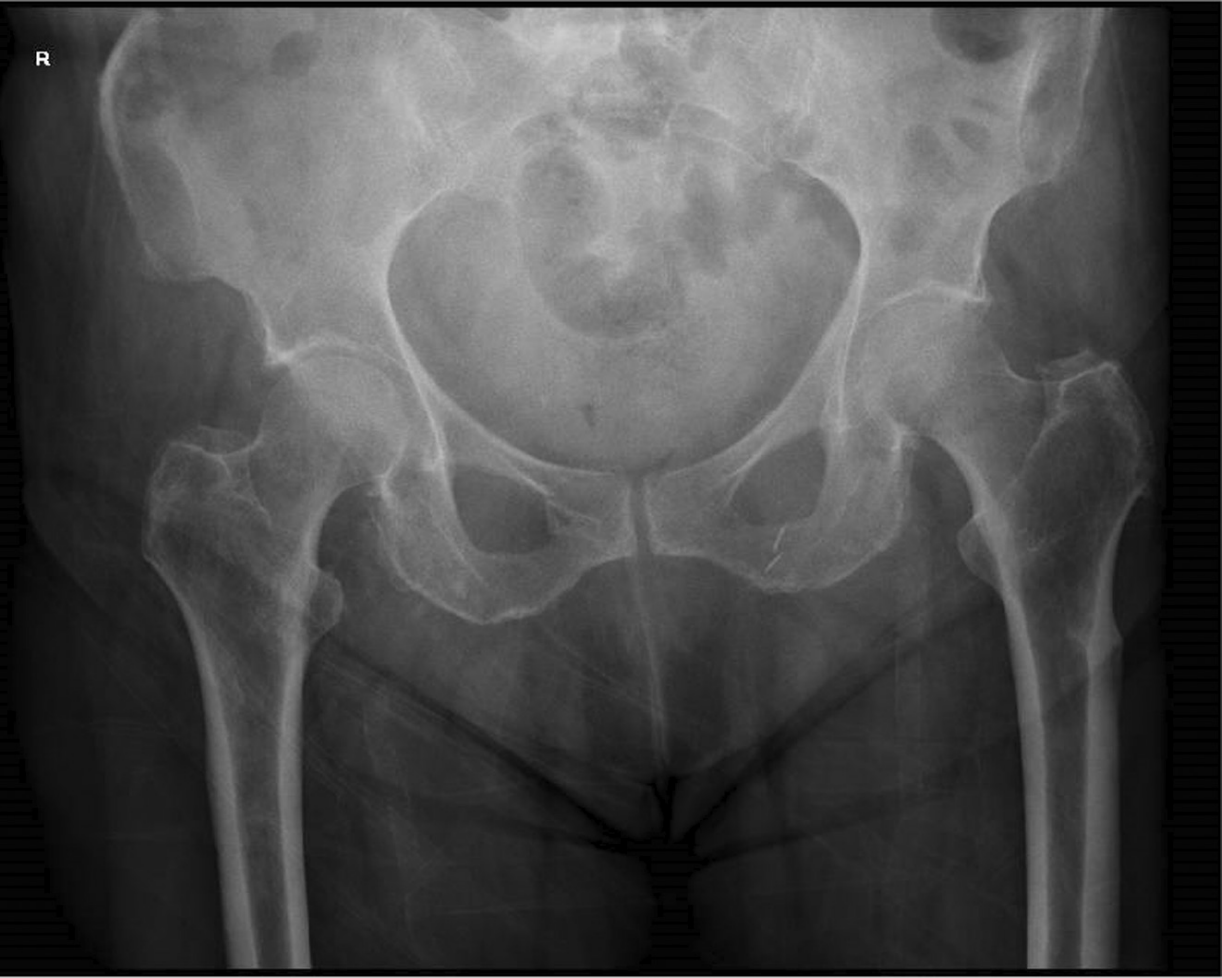
Pelvic Radiograph (23/4/17)—Cortical thickening in the lateral aspect of the left subtrochanteric region of the left femur, attributed to a possible ‘old fracture’, with subtle beaking of right proximal femoral lateral cortex.

**FIGURE 8:**
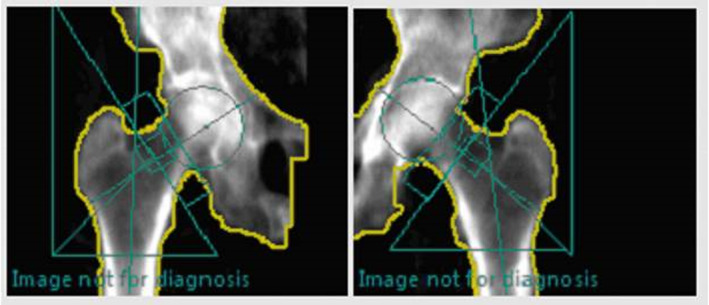
Breaking in the left femoral lateral cortex, 6 months prior to admission with a complete left AFF.

The patient sustained a fragility pelvic fracture 4 years prior to the current admission secondary to a fall. Radiographers queried a non‐acute, “healing fracture” of the left lateral proximal femur, and noted beaking on the right femoral lateral cortex (Figure 7). This demonstrates a missed opportunity for further patient's evaluation, investigation, appropriate cessation of BP therapy, and possible prophylactic treatment of what would eventually become the left acute AFF. The ASBMR suggest a 2–3 month trial of conservative therapy for asymptomatic patients with radiological evidence of impending AFF, after which prophylactic PFN is “strongly advised” (Table 2). The patients final DEXA scan, 6 months prior to admission (Figure 8) showed a further reduction in bone density, now meeting the criteria for osteopenia (left femur T‐score − 1.2, right femur −1.1, mean femur T‐score − 1.2).

As with any treatment, the risks and benefits should be analyzed, discussed with the patient, and changes in management made when indicated. Ultimately, AFFs are uncommon. With correct use and monitoring, BPs can prevent as many as 100 fractures for each adverse complication attributed to the treatment.^28^ This case demonstrates the value of DEXA scans identifying impending AFFs, up to 9 years before development of an acute complete fracture. If point‐of‐service femur imaging can provide an early indication of an incomplete AFF, then alternative preventative therapy can be initiated appropriately, and complete fractures can be prevented. Not only will this reduce morbidity associated with complete AFFs, but also reduce the burden of osteoporotic fractures in patients that stop BP therapy prematurely and without indication.

This highlights the importance of appropriate screening for impending AFFs. Both DEXA scans and plain X‐rays can reveal cortical thickening, stress changes in the femur, and possible fracture lines. If these exams are unremarkable but the patient remains symptomatic, further investigation with CT scan or MRI is advisable.^23^, ^29^ CT scans may be used for early detection of AFF,^30^ while MRI is recommended for follow‐up in cases of conservative management of AFF.^31^ Further research is needed to determine the most cost‐effective strategy for identifying this complication of osteoporosis treatment.^32^

Our case report has several limitations: the conclusions cannot be widely generalized or used to establish causation, the retrospective nature of the presentation, and the potential for reader distraction due to the focus on an unusual and rare case.

## 7 CONCLUSION/LEARNING POINTS

Effective screening for AFFs in patients on long‐term BPs is essential to reduce the risk of complications and mitigate the burden of osteoporosis and associated fragility fractures. DEXA scans can be utilized to identify early evidence of AFF, which, in our case, could be detected in an asymptomatic patient up to 9 years before the development of an acute complete fracture. Routine osteoporosis follow‐up and BP monitoring should include assessing for beaking or flaring of the lateral cortices. Clinicians must refer at‐risk patients for assessment and further management early enough to prevent avoidable complete fractures.

## AUTHOR CONTRIBUTIONS

**Ashraf Amin Ariff:** Conceptualization; data curation; methodology; project administration; resources; validation; writing – original draft. **Panagiotis Konstantinou:** Supervision; validation; writing – review and editing. **Michael Cuss:** Data curation; investigation; methodology; software; writing – original draft; writing – review and editing. **Charmaine Riley Nelson:** Writing – review and editing. **Ahmed Hamed:** Writing – review and editing. **Anastasios P. Nikolaides:** Conceptualization; data curation; investigation; methodology; supervision; validation; writing – original draft; writing – review and editing.

## FUNDING INFORMATION

The research received no funding grant.

## CONFLICT OF INTEREST STATEMENT

The authors declare they have no conflict of interest.

## ETHICS STATEMENT

All procedures performed in this study were in accordance of ethical standards of Helsinki declaration.

## CONSENT

Written informed consent was obtained from the patient next of kin (NOK) to publish this report in accordance with the journal's patient consent policy (as the patient deceased).

## Data Availability

Data sharing is applicable to this case report upon request.
